# Impact of Sarcopenia and Nutritional Status on Survival of Patients with Aortic Dissection: A Scoping Review

**DOI:** 10.3390/nu17193088

**Published:** 2025-09-28

**Authors:** Tomasz Semań, Sabina Krupa-Nurcek, Mateusz Szczupak, Jacek Kobak, Kazimierz Widenka

**Affiliations:** 1 Department of Surgery, Faculty of Medicine, Collegium Medicum, University of Rzeszów, 35-310 Warzywna, Poland; tseman@ur.edu.pl (T.S.); kwidenka@ur.edu.pl (K.W.); 2 Department of Anesthesiology and Intensive Care, Copernicus Hospital, 80-214 Gdańsk, Poland; szczupak.mateusz@icloud.com; 3 Department of Otolaryngology, Faculty of Medicine, Medical University of Gdańsk, 80-214 Gdańsk, Poland; jacek.kobak@gumed.edu.pl

**Keywords:** sarcopenia, nutritional status, aortic dissection, survival

## Abstract

Sarcopenia and the patient’s nutritional status play an important role in the course of many cardiovascular diseases, including aortic dissection. Disorders of muscle mass and function can affect the body’s ability to recover and the tolerance of surgical and pharmacological treatment. **Background/Objectives**: In patients with aortic dissection, the presence of sarcopenia may significantly worsen the prognosis and reduce the chances of survival. Weakening of muscle strength and metabolic disorders associated with poor nutrition negatively affect the body’s ability to regenerate after surgical interventions and conservative treatment. The aim of this review was to search the available literature on sarcopenia and nutritional status and their impact on mortality in patients with aortic dissection. **Methods**: This paper presents a scoping review and reviews the literature from the last 10 years. In the end, six articles which fit the criteria were included in this review. **Conclusions**: Studies indicate that the presence of sarcopenia correlates with a higher risk of complications and death, so the assessment of nutritional status and muscle function should be an integral part of the diagnosis and treatment of patients with aortic pathology. Nutritional assessment and early nutritional intervention are key to improving the prognosis and quality of life of these patients.

## 1. Introduction

Sarcopenia, defined as the progressive loss of muscle mass and strength, is a significant health problem in the aging population, especially among patients with cardiovascular disease [1,2,3]. In recent years, more and more attention has been paid to the interplay between sarcopenia and heart disease, pointing to their common pathophysiological mechanisms, such as chronic inflammation, oxidative stress, metabolic disorders and reduced physical activity [4,5]. The presence of sarcopenia in cardiac patients not only worsens the quality of life, but also increases the risk of hospitalization, complications and mortality. In the context of heart failure, coronary artery disease or hypertension, sarcopenia can be both an effect and a factor that exacerbates the course of the disease [4,6]. Skeletal muscle weakness leads to reduced exercise tolerance, which in turn hinders the implementation of cardiac rehabilitation and deepens social isolation. Moreover, changes in body composition, such as increasing fat mass while decreasing muscle mass, can affect the pharmacokinetics of drugs and the effectiveness of therapy [7,8].

Sarcopenia, although most commonly associated with aging and chronic metabolic diseases, can also occur in patients with aortic dissection, especially in its chronic phase [2,3]. People affected by this serious vascular pathology often require long-term hospitalization, restriction of physical activity and intensive pharmacological treatment, which promotes the loss of muscle mass and strength [9]. In addition, aortic dissection is most common in older people, in whom the risk of sarcopenia is already increased due to age and coexisting diseases such as hypertension or atherosclerosis. In the context of surgical treatment or conservative aortic dissection, the presence of sarcopenia may affect the prognosis, increasing the risk of complications and hindering rehabilitation [8,10]. Therefore, the assessment of the patient’s muscular condition should be an integral part of perioperative and long-term care, and early implementation of nutritional and physiotherapeutic interventions can improve treatment outcomes and quality of life of patients [11].

Aortic dissection is an emergency condition that requires urgent surgical intervention and carries a high risk of complications and mortality [12]. Recovery after aortic dissection surgery is a complex process that requires individually tailored rehabilitation and strict medical control [13]. In the first weeks of recovery, it is crucial to gradually increase physical activity, avoid excessive exertion and follow diet, pharmacotherapy and lifestyle recommendations. Rehabilitation should include low-intensity exercise to support the cardiovascular system and improve overall body performance, while monitoring blood pressure and heart function [13,14]. Psychological support and education of the patient and their relatives play an important role in adapting to life after surgery. Early implementation of a comprehensive rehabilitation program can significantly improve quality of life, reduce the risk of relapse and support a full return to daily activities [3]. Sarcopenia is compared to other geriatric syndromes due to its direct dependence on changes in the musculoskeletal system, and not only on psychosocial or metabolic factors. Unlike frailty syndrome, which encompasses a wider spectrum of symptoms such as decreased physical performance, weight loss, or decreased activity, sarcopenia focuses mainly on muscle quality and function. Moreover, its diagnosis is based on objective measurements of muscle strength and muscle mass, which allows for precise differentiation from other conditions, such as malnutrition or depression, which can co-occur but have different pathophysiological mechanisms [10,13].

**Figure 1:**
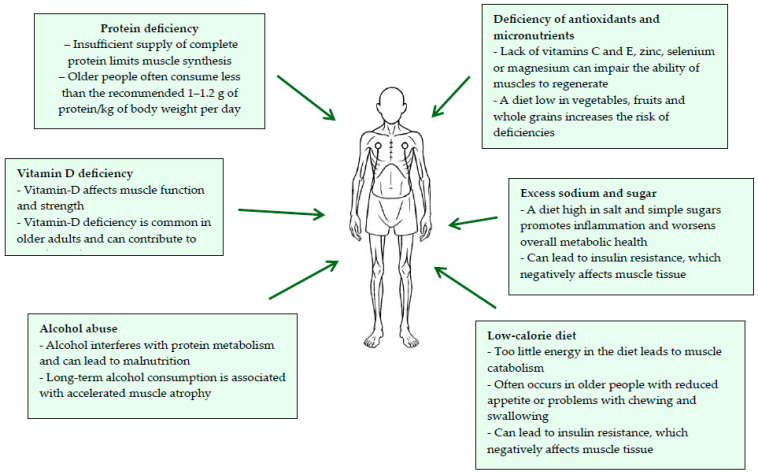
Nutrition-related factors influencing the development of sarcopenia in patients after heart surgery. Source: Authors’ own work.

Proper nutrition after aortic dissection surgery plays a key role in the recovery process and prevention of complications, including the development of sarcopenia [3,15]. In the postoperative period, the body has an increased demand for nutrients, especially wholesome protein, which supports tissue regeneration and the maintenance of muscle mass. It is recommended to consume 1.5–2 g of protein per kilogram of body weight per day, coming from lean meat, fish, eggs, dairy products and legumes [16,17]. At the same time, highly processed foods, fatty foods and products that negatively affect the metabolism of drugs, such as grapefruit, should be avoided [18]. Dietary support, preferably under the supervision of a specialist, allows the adjustment of the menu to the individual needs of the patient, taking into account their clinical condition, medications taken and level of physical activity. Proper nutrition, combined with physical rehabilitation, is the foundation of sarcopenia prevention and promotes faster recovery after aortic surgery [16,19,20]. Nutrition-related factors influencing the development of sarcopenia in patients after heart surgery are presented on the Figure 1.

### Objectives and Rationale

The aim of this review was to search the available literature on sarcopenia and nutritional status and their impact on mortality in patients with aortic dissection. Of particular interest were the factors that play a key role in the occurrence of sarcopenia, the nutritional status and the impact of these factors on the survival of patients undergoing cardiac surgery. The review question (RQ) for our scoped review is as follows: Does sarcopenia and nutritional status have an influence on the survival of patients with aortic dissection? The aim of the authors was to identify nutritional factors and factors associated with sarcopenia in order to analyze the survival of patients after aortic dissection.

## 2. Materials and Methods

### 2.1. Study Design

We chose the scope review method because we wanted to create a map of concepts relevant to the phenomenon of sarcopenia in patients after aortic dissection. Scoping reviews are a relatively new methodological approach. Currently, there is little guidance on choosing between a systematic review and a scoping review when synthesizing evidence, especially when the literature has not yet been comprehensively reviewed or is large, complex, or heterogeneous in nature and cannot be amenable to a more detailed systematic review [21]. We have prepared the scope review in accordance with the methods described in the Joanna Briggs Institute’s methodological manual for scope reviews and using the recommendations contained in the Preferred Reporting Items for Systematic Reviews and Meta-analysis for Scoping Reviews (PRISMA-ScR) guidelines [22,23].

### 2.2. Inclusion and Exclusion Criteria

To identify important aspects related to the phenomenon of sarcopenia and nutritional status of patients after aortic dissection and mortality in this group, we developed a research question that clearly defined the population, concept and context of the review scope.

The inclusion criteria were as follows:

- Articles published in 2015–2025;
- Original articles (observational and randomized trials), meta-analyses, systematic and narrative reviews;
- Articles with access to the full text;
- English-language articles.

The exclusion criteria included the following:

- Publications older than 10 years;
- Case reports, comments, letters to the editor, book chapters;
- No full-text article;
- Articles in a language other than English.

*Population*

The review included studies describing sarcopenia, nutritional status and mortality in patients with aortic dissection. In this review, sarcopenia was defined as a progressive condition characterized by the age-related loss of muscle mass, strength and function. Aortic dissection is defined as a serious condition in which a tear occurs in the inner layer of the body’s main artery (aorta). Nutritional level is the quality or quantity of nutrients in food, which indicates how well it can contribute to an individual’s health and sustenance [2,3,13,16].

*Concept*

The focus was on mortality and the prevalence of sarcopenia and nutritional status in patients with aortic dissection. This study aimed to investigate the impact of sarcopenia and nutritional status on mortality in patients with aortic dissection.

*Context*

The studies to be included in the review included those on patients with aortic dissection.

*Types of studies*

This review included a retrospective observational study of any design or methodology.

### 2.3. Search Strategy

Three authors searched the following databases: PubMed, Scopus, EBSCO, Web of Science, Google Scholar, and the Cochrane Library. The Mozilla Firefox search engine was used for searching. The following keywords were used: “sarcopenia”, “aortic dissection”, “cardiac surgery”, “sarcopenia after aortic dissection”, “sarcopenia in cardiopulmonary surgery”, “nutrition in aortic dissection”, “sarcopenia and nutritional”, “mortality and sarcopenia and aortic dissection”. Many studies addressed the incidence of sarcopenia after cardiac surgery after valve repair, which was not the purpose of this review. We entered keywords and their combinations using AND or OR. All publications were screened by title and abstract to exclude irrelevant records. Any discrepancies were resolved through discussion with five investigators, and at the end of the selection process, full agreement was reached on the articles to be included. The initial search ran from inception to 19 May 2025, and the final search was conducted on 20 July 2025.

### 2.4. Extraction of Data

A data extraction form based on the JBI scoping review guidelines [22] was used and key information from the studies was included. Data extraction, which is referred to as “data plotting” in the scoping review [24], was performed by two independent reviewers. To identify relevant studies, we used the Population-Concept-Context (PCC) framework. Information extracted from the studies included the following: first author, year, country, study design, study objective, inclusion and exclusion criteria (PCC), outcomes, and findings. The authors performed the extraction using Microsoft Excel.

### 2.5. Critical Appraisal Process

A scoping review may include a review of current evidence without including a methodological assessment of the included studies [22].

### 2.6. Process for Including Publications in the Review

**Figure 2:**
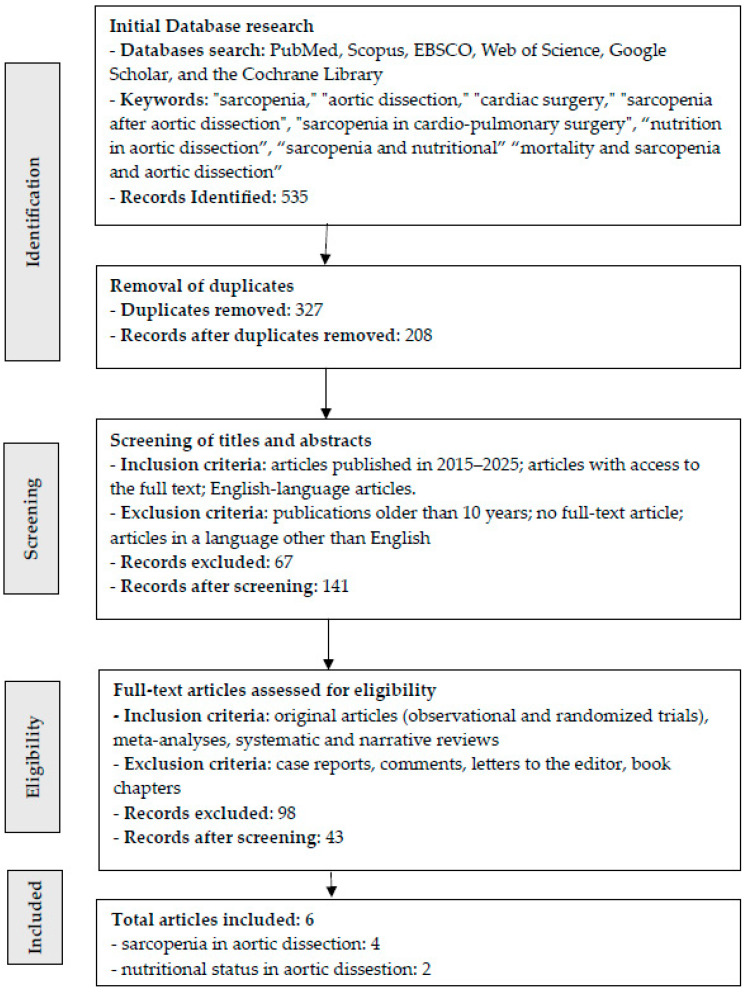
Literature search and selection flowchart for this review.

In our coverage review, we identified originally 535 articles, of which 4 on the occurrence of sarcopenia in patients with aortic dissection were ultimately included (Figure 2). The other 2 articles about nutrition in sarcopenia in aortic dissection were included in this review. After removing duplicates (*n* = 327), 208 articles remained. After reviewing the articles according to the inclusion and exclusion criteria (*n* = 67), 141 articles remained. Ninety-eight publications lacked full text and were excluded, leaving 43 papers. As a result, after meeting all requirements, six publications were included in this review. Among the included studies, all were retrospective observational studies. The research was conducted in Korea (*n* = 1), USA (*n* = 1), Japan (*n* = 1), and China (*n* = 3). The studies included in this review used CT scans of patients to assess sarcopenia and nutritional assessment was conducted by using research tools. Survival assessment was statistically assessed. The results are presented in Table 1.

## 3. Factors Influencing the Development of Sarcopenia in Patients with Aortic Dissection

### 3.1. Obesity

In recent years, more and more attention has been paid not only to factors directly related to the pathogenesis of dissection, such as hypertension or connective tissue diseases, but also to the general condition of the patient—including their body weight, body composition and muscle function. The phenomenon of the coexistence of obesity and sarcopenia, referred to as “sarcopenic obesity”, which can significantly affect the course of the disease, prognosis and effectiveness of treatment, is gaining particular importance [31,32,33]. The concept of the “obesity paradox” may be applied in particular to the older population; therefore, the impact of obesity on cardiovascular disease and mortality remains unclear [34,35]. Therefore, the epidemiological estimate of sarcopenic obesity remains imprecise and its prevalence varies depending on the definition used [36]. Dutch Lifelines study data from 18–90-year-olds showed a global prevalence of sarcopenic obesity of 1.4% in women and 0.9% in men, respectively, with an increase in prevalence in the age of 50 and a prevalence of up to 16.7% in the 80–89 age group [37]. A meta-analysis of 50 studies representing 86,285 people found an incidence of 11% of sarcopenic obesity in adults aged 60 years [38]. Sarcopenic obesity is a more serious complication than obesity itself. Sarcopenic obesity is associated with a higher risk of many disorders, such as cardiovascular disease, and faster death [39,40]. Cardiovascular disease (CVD) is the leading cause of death in Western countries. This condition has recently been linked to the occurrence of sarcopenic obesity [41,42]. Kays et al. describe that both overweight and non-overweight patients may manifest sarcopenia [26].

Obesity is accompanied by chronic inflammation, which is induced by pro-inflammatory cytokines (m.in. TNF-α, IL-6) and leads to disorders in muscle metabolism [43,44]. Muscle protein synthesis is impaired, their degradation is exacerbated and insulin sensitivity is reduced, which in turn leads to loss of muscle mass and strength—i.e., sarcopenia [45,46]. Obese people often experience reduced physical activity, which further exacerbates muscle atrophy. Importantly, in such patients, body weight may mask muscle loss, which makes it difficult to diagnose sarcopenia and delays the implementation of appropriate interventions [46,47,48]. In patients with aortic dissection, obesity is a risk factor not only for the vascular event itself, but also for perioperative complications [49]. Excessive body weight makes surgical access difficult, increases the risk of infection, wound-healing disorders and thromboembolic complications. At the same time, if sarcopenia coexists in such a patient, his metabolic reserves are significantly limited, which impairs the ability to regenerate and adapt after surgery [47,49]. Sarcopenic obesity is particularly dangerous because it combines the negative effects of both conditions—chronic inflammation, insulin resistance, lipid disorders and muscle weakness [41,45,46]. Such patients have worse rehabilitation outcomes, longer hospitalization and a higher risk of recurrence and death. Patients with aortic dissection, obesity and sarcopenia require the care of a team of specialists—vascular surgeons, cardiologists, dieticians, physiotherapists and psychologists [45,46]. Only a comprehensive approach allows for effective treatment, minimizing the risk of complications and improving the quality of life. Patient education, motivation to change lifestyle and regular monitoring of clinical parameters are indispensable elements of therapy [48,49].

Included in this review, the article by Kays et al. describes that sarcopenia combined with obesity increases the risk of mortality by 3.5 times compared to obese patients without sarcopenia. According to the calculations of the authors of this work, patients with sarcopenia had a shorter survival time compared to patients without sarcopenia [26]. Age, congestive heart failure, cerebrovascular disease and diabetes have been shown to increase the risk of mortality after aortic dissection surgery [50]. Based on the results of these studies, it can be concluded that every patient qualified for aortic repair surgery should be tested for the presence of sarcopenia. Examinations should be carried out both before and after surgery. Investigating the risk of developing sarcopenia may be beneficial for monitoring the patient’s nutritional parameters. Nutritional status has a great effect on the recovery of patients after aortic dissection surgery [51,52,53].

### 3.2. Age

Aging of the body is an inevitable process that affects all systems and organs, including the cardiovascular and musculoskeletal systems [54]. In the context of aortic dissection—one of the most severe and dramatic vascular pathologies—the patient’s age plays a key role not only as a risk factor for the event itself, but also as a determinant of the clinical course, prognosis, and ability to regenerate [55]. One of the most important phenomena accompanying aging is sarcopenia—a progressive loss of muscle mass and function, which can significantly worsen the patient’s general condition, increase the risk of complications and hinder the treatment process [39,41,43]. Sarcopenia is recognized as one of the major geriatric syndromes, along with frailty syndrome, osteoporosis and fall syndrome [56]. With age, there is a decrease in the number and size of muscle fibers, especially type II (fast-twitch), a decrease in the activity of anabolic hormones such as testosterone, growth hormone or IGF-1, an increase in low-intensity inflammation (“inflammaging”), which promotes muscle catabolism, a decrease in physical activity and mobility, a deterioration in nutrient absorption and nutritional deficiencies [57,58,59]. As a result, older people experience a gradual loss of strength, muscle mass, and physical fitness, which can lead to disability, an increased risk of falls, and, in the context of acute diseases, a worse prognosis [60,61]. Aortic dissection is most common in people over 60 years of age, although it can also occur earlier, especially in the course of connective tissue diseases (e.g., Marfan syndrome). In elderly patients, the clinical picture is less typical, and symptoms may be masked by concomitant diseases. Importantly, age affects the structure of the aortic wall—it reduces its elasticity, increases rigidity and susceptibility to mechanical damage [62]. In the case of surgical intervention in elderly patients, the risk of complications is higher and the ability to regenerate is limited. Sarcopenia, as a common companion of aging, further worsens the prognosis, increasing the risk of respiratory failure, infection, wound-healing disorders and prolonging the time of hospitalization [62,63,64]. Treatment of an elderly patient with aortic dissection and sarcopenia requires the cooperation of many specialists—vascular surgeons, geriatricians, dieticians, physiotherapists and nurses [65]. Only a comprehensive approach allows for effective treatment, minimizing the risk of complications and improving physical function. Education of the patient and his family, motivation to be active and regular monitoring of clinical parameters are indispensable elements of therapy [66]. As a result, older people experience a gradual loss of strength, muscle mass and physical fitness, which can lead to disability, an increased risk of falls, and, in the context of acute diseases, a worse prognosis [65,66,67]. Ishigaki et al. described in their paper that the risk of death in patients with sarcopenia increases with age. The risk of death of patients with sarcopenia is higher at 85 years of age or older [27].

### 3.3. Comorbidities

Comorbidities can influence the development of sarcopenia through a variety of mechanisms. An analysis of the relationship between the number of chronic diseases and sarcopenia proved that the risk of sarcopenia with four to five chronic diseases was 1.8 times higher [68]. Chronic diseases such as type 2 diabetes, chronic kidney disease or autoimmune diseases are associated with persistent, low-grade inflammation [69]. Pro-inflammatory cytokines (e.g., TNF-α, IL-6) enhance muscle catabolism and inhibit muscle protein synthesis. Insulin resistance, hyperglycemia or dyslipidemia have a negative effect on muscle metabolism, leading to muscle weakness and atrophy [69,70,71]. Gastrointestinal diseases, cancers or chronic infections can lead to malnutrition, deficiencies of protein, vitamins and microelements, which directly affects muscle mass. Musculoskeletal diseases (e.g., osteoarthritis), chronic obstructive pulmonary disease (COPD) or heart failure limit the ability to move, which promotes muscle wasting. The use of many drugs, especially glucocorticoids, statins or diuretics, can negatively affect muscle metabolism and accelerate the development of sarcopenia [72,73,74].

Patients with aortic dissection often suffer from hypertension, atherosclerosis, diabetes, chronic kidney disease, COPD or heart disease [75]. Each of these diseases can contribute to the development of sarcopenia, both by direct action on the muscles and by limiting physical activity, deterioration of nutritional status or intensification of inflammation [74,75,76]. For example, hypertension is associated with vascular damage and decreased muscle perfusion, which can lead to hypoxia and weakness. Type 2 diabetes leads to muscle insulin resistance, disorders in glucose and protein metabolism, and increases the risk of neuropathy, which limits muscle function. Chronic kidney disease, on the other hand, is associated with electrolyte imbalances, metabolic acidosis and vitamin D deficiency—all of which promote muscle loss [68,69,70,71]. Chronic hypoxemia and hypercapnia negatively affect the respiratory and peripheral muscles, and limiting physical activity accelerates the development of sarcopenia [73,74,75,76].

## 4. Nutritional Status of Patients with Aortic Dissection

Aortic dissection is an emergency condition that requires prompt surgical intervention and is associated with a high risk of mortality and complications. Among patients affected by this condition, concomitant sarcopenia is increasingly observed [77]. In this context, adequate nutrition before and after surgery becomes crucial, not only as an element supporting convalescence, but also as a factor influencing survival and quality of life of the patient [77,78,79,80]. Sarcopenia, although originally associated with the aging process, is increasingly diagnosed in patients with chronic and acute diseases, including aortic dissection. Loss of muscle mass leads to a decrease in the body’s ability to cope with the metabolic stress associated with surgery [81,82]. Hypercatabolic syndrome occurs in the early stages of aortic dissection and can balance the nutritional status of patients, affecting their prognosis [83,84].

Weakening of the respiratory muscles increases the risk of pulmonary complications, and reduced muscle strength negatively affects mobility and the ability to undergo postoperative rehabilitation [85]. Sarcopenia is also an independent risk factor for mortality, making it an important component of preoperative assessment [74,86,87]. Preoperative assessment of nutritional status should be comprehensive and take into account both anthropometric (body weight, BMI, arm circumference) and biochemical parameters (albumin, prealbumin, CRP), as well as functional parameters (handshake strength, performance tests) [85,86,87,88,89]. In patients with aortic dissection, due to the urgency of the intervention, the time for a full assessment may be limited, but even baseline indicators can provide valuable information. If sarcopenia is suspected, it is worth considering the use of tools such as SARC-F or assessment of muscle mass using bioimpedance [90,91]. Ideally, nutritional interventions should begin a few days before surgery. In practice, with aortic dissection, this time is often reduced to a minimum. Nevertheless, even short-term nutritional support can be beneficial. It is recommended to use oral dietary supplements (ONS) rich in protein, leucine, vitamin D and omega-3 fatty acids, which support muscle protein synthesis and have anti-inflammatory effects [89,90,91,92]. In cases where oral feeding is not possible, enteral or parenteral nutrition should be considered, in accordance with current ESPEN guidelines [93]. After aortic surgery, patients often require intensive medical care, and their metabolic state is significantly strained. Early initiation of nutrition—preferably within 24–48 h after surgery—is crucial for reducing muscle catabolism [87,89]. In the first days after surgery, enteral nutrition is preferred, which supports the function of the intestinal barrier and reduces the risk of infection. As the clinical condition improves, the transition to oral nutrition should be continued, continuing with protein and energy supplementation. In the case of patients with sarcopenia, special attention should be paid to protein supply—1.2–1.5 g/kg body weight/day is recommended, taking into account high-quality sources of amino acids [68,92]. Leucine, as a key anabolic amino acid, should be supplied in an amount of at least 2–3 g per serving of protein. Additionally, supplementation with vitamin D (especially in people with a deficiency) and omega-3 may support muscle recovery and reduce inflammation [94]. The patient’s nutritional status should be monitored throughout hospitalization and convalescence. Regular assessment of body weight, biochemical parameters and muscle function allows for the adaptation of nutritional therapy to changing needs [84,85,86]. Low food intake and monotonous diets put older adults at risk of insufficient nutrient intake [94,95]. In the absence of improvement, intensifying nutritional support or modifying the composition of supplements should be considered.

In emergency patients who require urgent surgery, nutritional interventions focus on rapid metabolic support and minimizing the risk of malnutrition [91,93,94]. Depending on the clinical condition, enteral or parenteral nutrition is used, with enteral nutrition being preferred if the functioning of the gastrointestinal tract allows it. It is crucial to provide the right amount of energy, protein and micronutrients to support tissue healing, maintain immune function and reduce postoperative complications. In clinical practice, nutrition protocols are often implemented in accordance with guidelines, taking into account the individual needs of the patient and the dynamics of their health [93,94].

## 5. Survival of Patients with Aortic Dissection Including Sarcopenia and Nutritional Status

In the context of aortic dissection, the presence of sarcopenia can significantly worsen the prognosis. Patients with reduced muscle mass show lower metabolic reserve, weaker immune response and limited ability to hemodynamic compensation [96]. In practice, this means that people with sarcopenia are more likely to suffer from shock, multi-organ failure, and postoperative complications such as infections, respiratory failure or thrombosis [97]. In addition, sarcopenia often coexists with other risk factors—such as malnutrition, diabetes, chronic kidney disease and hypertension—which in themselves increase the risk of death in the course of aortic dissection. Sarcopenia in geriatric patients increased the length of hospital stay and mortality [98]. Clinical studies indicate that patients with aortic dissection and concomitant sarcopenia have a significantly higher mortality rate in both acute and long-term follow-up. In one retrospective analysis, it was shown that the presence of sarcopenia increases the risk of death after aortic repair surgery by up to 30–40%, regardless of the type of dissection [99]. What is more, these patients more often require longer hospitalization, intensive care and rehabilitation, and their return to independence is significantly more difficult. It is also worth noting that sarcopenia can influence therapeutic decisions [100]. Surgeons and interventional cardiologists, when assessing surgical risk, increasingly take into account the patient’s muscle status as one of the key parameters. In borderline cases, the presence of sarcopenia may prompt the therapeutic team to opt for less invasive treatments or even to abandon surgical intervention in favor of conservative treatment, which may also affect the final prognosis [100,101,102]. From the point of view of prevention and patient care, it is crucial to diagnose sarcopenia early and implement measures to improve muscle mass and strength. Nutrition programs, protein supplementation, resistance exercise and monitoring of the patient’s functional status should be an integral part of the care of people with cardiovascular diseases, especially those who are at risk of aortic dissection [103,104]. Involving a rehabilitation team, dietician and geriatrician in the treatment process can significantly improve clinical outcomes and reduce the risk of death. In conclusion, sarcopenia is a significant, though often underestimated, risk factor for mortality in patients with aortic dissection. Its presence worsens the prognosis, increases the risk of complications and affects therapeutic decisions [104,105,106]. An integrated approach to treatment, taking into account both vascular and systemic aspects, is essential to improve survival and quality of life in these patients. In the era of an aging population and a growing number of people with chronic diseases, this topic deserves special attention in clinical practice and scientific research [96,100,101,102].

Sarcopenia may be a useful predictor of mortality, especially in populations of the elderly and patients with chronic diseases [97,98]. Sarcopenia, defined as the progressive loss of muscle mass and strength, is not only a physiological symptom of aging, but is increasingly treated as an independent risk factor for deterioration, disability, hospitalization and death. Numerous observational studies and meta-analyses have shown that people diagnosed with sarcopenia have a significantly higher risk of mortality compared to people with preserved muscle mass [98,99]. This applies to both general mortality and deaths related to specific disease entities, such as cardiovascular diseases, cancer or infections. The mechanisms that explain this relationship are multifactorial. Muscle weakness leads to reduced mobility, an increased risk of falls and fractures, as well as a deterioration in respiratory and immune function [107]. Sarcopenia often coexists with malnutrition, chronic inflammation and frailty syndrome, which further increases the body’s susceptibility to physiological stressors such as surgery, infections or exacerbations of chronic diseases. In clinical practice, this means that patients with sarcopenia tolerate treatment less well, have a longer recovery time and more often require institutional care [107,108,109]. Importantly, sarcopenia can be detected relatively easily using simple screening tools, such as the assessment of handshake strength, walking speed or the SARC-F questionnaire [110]. This makes it possible to identify high-risk patients early and implement interventions—both nutritional and physiotherapeutic—that can improve the prognosis. In the context of predicting mortality, the presence of sarcopenia should be treated as an alarm signal, requiring a comprehensive assessment of the health condition and adjustment of the treatment plan [110,111,112]. Incorporating muscle mass assessment into standard diagnostic procedures can significantly increase the accuracy of clinical prognosis and improve the quality of patient care [99]. According to Lin et al., the Prognostic Nutritional Index (PNI), age, hypertension and post-operative stay time affected hospital mortality, and a low PNI was associated with poorer prognosis of patients after aortic dissection surgery [26]. Myosteatosis is a pathological accumulation of fat in muscle tissue, which leads to a deterioration in its quality and function, despite the preserved muscle mass [113]. This phenomenon is particularly important in patients with a life-threatening condition, where metabolic disorders, oxidative stress and chronic inflammation promote muscle fat infiltration. Myosteatosis may affect a worse prognosis, an increased risk of postoperative complications and a longer recovery time, which is why its early diagnosis and inclusion in the assessment of the patient’s nutritional status is of key importance in intensive care [113]. To quantify the area of VFA, SFA and skeletal muscles, axial CT images at the level of the third lumbar vertebra (L3) are analyzed using a semi-automatic method using a special viewer [114]. Summary of key factors influencing the development of sarcopenia in patients with aortic dissec-tion are presented in the Table 2. Comparison and differences in sarcopenia and myosteatosis are presented in the Table 3.

The Table 4 shows the definitions of SMI and PMI (L3) in the context of myosteatosis.

## 6. Limitations and Future Research

Studies on nutritional status and sarcopenia in patients with aortic dissection face a number of important limitations, both methodological and clinical. Aortic dissection is an emergency, making it difficult to make a full assessment of the patient’s nutritional status and muscle mass at the time of admission to the hospital. There is a lack of standardized tools for rapid and reliable assessment of sarcopenia in acute care. Most of the available methods (e.g., computed tomography, DEXA, bioimpedance) require a stable patient’s condition, which is not always possible. Patients with aortic dissection are often unable to cooperate with functional tests, such as grip force measurements or physical fitness tests. There are no unambiguous standards for the assessment of sarcopenia in the population of patients with acute vascular diseases. It is difficult to determine whether sarcopenia is a risk factor, an effect of the disease or an independent concomitant phenomenon. Despite these difficulties, there is a growing awareness of the importance of assessing the state of nutrition and muscle mass in the context of prognosis and rehabilitation planning. This is an area that definitely needs more research and better diagnostic tools.

It should be noted that the available studies are usually conducted on a small group; statistical errors may occur. In addition, treatment departments should be adapted with tools to assess the risk of developing sarcopenia. Each department should accurately assess the patient’s nutrition, take into account the results of laboratory tests (m.in. protein level) and assess the patient’s frailty. Procedures should be implemented that will be responsible for the use of standardized tools to assess the level of sarcopenia in patients in an emergency. Other limitations of this review are that all studies were retrospective, small cohorts, heterogeneous definitions of sarcopenia, variability in CT thresholds, and with no formal assessment of risk of error. The publications included in this review did not have methodological homogeneity (SMI/PMI, radiodensity) and had potential selection errors. In the future, it is also necessary to separate the works related to abdominal aortic aneurysm, creating similar reviews. Currently, there are too few studies and therefore studies on the abdominal aorta have been included in this review. Directions for future research should include prospective cohorts in aortic dissection, standardization of CT measurements, inclusion of objective functional tests (e.g., handshake strength) and validation of PNI/GNRI thresholds.

## 7. Conclusions

Sarcopenia, i.e., the progressive loss of muscle mass and strength, is a significant clinical problem in patients with aortic dissection, especially in the elderly. Long-term hospitalization, limitation of physical activity and metabolic stress associated with the acute course of the disease promote the development of malnutrition and muscle catabolism. In addition, the presence of inflammation, hormonal disorders and polypharmacotherapy exacerbates the risk of sarcopenia. Nutritional assessment and early nutritional intervention are key to improving the prognosis and quality of life of these patients.

## Figures and Tables

**Table 1:** Characteristics and findings of studies included in this review.

Author, Year	Country	Participants	Findings
Bom J.S. et al., 2025 [25]	Korea	Patients with aortic dissection	✓ Early mortality did not differ significantly between the sarcopenia and non-sarcopenia groups, late mortality was significantly higher in the sarcopenia group ✓ Age and presence of sarcopenia have been identified as independent predictors of late mortality ✓ The presence of sarcopenia does not affect early mortality after surgery ✓ Sarcopenia is associated with late mortality
Kays J.K. et al., 2019 [26]	USA	Patients with aortic dissection	✓ More than half of all patients with aortic dissection have sarcopenia ✓ Sarcopenia with myosteatosis together are greater predictors of mortality compared to sarcopenia alone ✓ A total of 22% of the subjects showed neither sarcopenia nor myosteatosis ✓ Both overweight and non-overweight patients may manifest sarcopenia ✓ The presence of sarcopenia is a risk factor independent of other known risk factors ✓ Sarcopenia combined with obesity increases the risk of mortality by 3.5 times compared to obese patients without sarcopenia
Ishigaki T. et al., 2023 [27]	Japan	Patients with aortic dissection	✓ Mortality was higher in the group of patients with sarcopenia ✓ Risk of death increases in patients with sarcopenia with age ✓ A group of patients with sarcopenia had a lower rate of discharge from home than patients without sarcopenia
Lin X. et al., 2024 [28]	China	Patients with aortic dissection	✓ Sarcopenia significantly affected long-term survival ✓ Sarcopenia was an independent predictor for reduced survival ✓ Sarcopenia may be a useful tool for predicting short- and long-term mortality in patients after aortic dissection surgery
Lin Y. et al., 2021 [29]	China	Patients with aortic dissection (type A)	✓ Prognostic Nutritional Index (PNI), age, hypertension and length of stay in the ward after surgery affected hospital mortality ✓ Low PNI was associated with poorer prognosis of patients after aortic dissection surgery
Lin L. et al., 2025 [30]	China	Patients with aortic dissection	✓ Geriatric nutritional risk index (GNRI), age, WBC, platelet count, creatinine level, hs-TNT, LVEF—50%, CPB time and lactate levels have been identified as independent risk factors for in-hospital mortality

**Table 2:** Summary of key factors influencing the development of sarcopenia in patients with aortic dissection.

Factor	Key Findings	References
Obesity	According to the literature, both overweight and obese patients can manifest sarcopenia. Sarcopenia combined with obesity increases the risk of mortality by 3.5 times compared to obese patients without sarcopenia. Sarcopenic obesity is more dangerous for patients with aortic dissection compared to obesity alone.	[26,31,32,33,34,35,36,37,38,39,40,41,42,43,44,45,46,47,48,49,50,51,52,53]
Nutritional status	Hospitalization, metabolic stress and dietary restrictions can lead to protein and calorie deficiencies. Malnutrition is one of the main factors in the development of sarcopenia.	[30,68,77,78,79,80,81,82,83,84,85,86,87,88,89,90,91,92,93,94,95]
Comorbidities	Hypertension, diabetes, heart failure—common in patients with aortic dissection—further increase the risk of sarcopenia. In addition, other diseases affect the prolongation of recovery in patients with aortic dissection.	[68,69,70,71,72,73,74,75,76]
Age	With age, the risk of nutritional deficiencies and cardiovascular diseases increases, and as a consequence, sarcopenia develops. Aortic dissection is also more common in patients over 60 years of age.	[25,27,28,30,54,55,56,57,58,59,60,61,62,63,64,65,66,67]

**Table 3:** Sarcopenia vs. myosteatosis—comparison and differences [31,32,33,113,114].

Feature	Sarcopenia	Myosteatosis
Definition	Progressive loss of muscle mass, strength and function mainly associated with age	Pathological fat deposition in muscle tissue
Main Cause	Aging, physical inactivity, hormonal deficiencies	Metabolic disorders, obesity, insulin resistance
Clinical signs	Weakness, difficulty moving, increased risk of falls	Reduced muscle quality, reduced strength despite retained muscle mass
Changes in muscle tissue	Reduction in the number and size of muscle fibers	Accumulation of fat inside and between muscle fibers
Diagnostics	Muscle mass measurement, strength and fitness tests	Imaging (MRI, CT) showing fat in muscle
Health effects	Increased risk of disability, osteoporosis, hospitalization	Deterioration of muscle function, risk of insulin resistance and type 2 diabetes
Treatment	Resistance exercises, protein diet, supplementation, hormone therapy	Weight reduction, physical activity, improved metabolic control

**Table 4:** Definitions of SMI and PMI (L3) in the context of myosteatosis [113,114,115].

SMI (Skeletal Muscle Index)	PMI (Psoas Muscle Index)
is an index of skeletal muscle mass, calculated as the muscle surface area (in cm^2^) at the level of the third lumbar vertebra (L3) divided by height squared (m^2^)	is a similar indicator, but based solely on the area of the greater lumbar muscle (psoas major) at the L3 level, also divided by the height squared.
in the context of myosteatosis, SMI is used to assess the quantity of muscle, but not its quality—which is why it is often combined with the measurement of fatty muscle infiltration (e.g., by the value of attenuation in CT).	PMI is sometimes used as a simplified marker of muscle mass, especially in research on osteoporosis and fracture risk

In the case of myosteatosis, not only the amount of muscle (SMI/PMI) is crucial, but also its quality—that is, the degree of fat infiltration. Therefore, the study also uses Muscle attenuation (MA)—the value of muscle attenuation in computed tomography (CT), expressed in Hounsfield units (HU). Lower HU values indicate a higher fat content in the muscles, i.e. the presence of myosteatosis.

## Data Availability

Not applicable.

## Abbreviations

The following abbreviations are used in this manuscript:

RQ	review question
PRISMA-ScR	Preferred Reporting Items for Systematic Reviews and Meta-analysis for Scoping Reviews
PNI	Prognostic Nutritional Index
GNRI	Geriatric Nutritional Risk Index
MRI	Magnetic resonance imaging
CT	Computed tomography
